# Nocturnal to Diurnal Switches with Spontaneous Suppression of Wheel-Running Behavior in a Subterranean Rodent

**DOI:** 10.1371/journal.pone.0140500

**Published:** 2015-10-13

**Authors:** Patricia Tachinardi, Øivind Tøien, Veronica S. Valentinuzzi, C. Loren Buck, Gisele A. Oda

**Affiliations:** 1 Instituto de Biociências, Universidade de São Paulo, São Paulo, Brazil; 2 Institute of Arctic Biology, University of Alaska, Fairbanks, United States of America; 3 Centro Regional de Investigaciones Científicas y Transferencia Tecnológica (CRILAR), La Rioja, Argentina; 4 Department of Biological Sciences, University of Alaska, Anchorage, United States of America; Kent State University, UNITED STATES

## Abstract

Several rodent species that are diurnal in the field become nocturnal in the lab. It has been suggested that the use of running-wheels in the lab might contribute to this timing switch. This proposition is based on studies that indicate feed-back of vigorous wheel-running on the period and phase of circadian clocks that time daily activity rhythms. Tuco-tucos (*Ctenomys* aff. *knighti*) are subterranean rodents that are diurnal in the field but are robustly nocturnal in laboratory, with or without access to running wheels. We assessed their energy metabolism by continuously and simultaneously monitoring rates of oxygen consumption, body temperature, general motor and wheel running activity for several days in the presence and absence of wheels. Surprisingly, some individuals spontaneously suppressed running-wheel activity and switched to diurnality in the respirometry chamber, whereas the remaining animals continued to be nocturnal even after wheel removal. This is the first report of timing switches that occur with spontaneous wheel-running suppression and which are not replicated by removal of the wheel.

## Introduction

The tuco-tuco (*Ctenomys* aff. *knighti*), a South American subterranean rodent, is among the several mammals described as having discrepant activity timing between field and laboratory conditions [1–7]. Whereas they are active during the day in semi-natural field enclosures, all individuals are nocturnal under laboratory conditions, with or without access to running-wheels [8–10] suggesting that a fundamental feature of their natural environment is not reproduced in the laboratory. Both ecological and physiological studies indicate the critical role of daily energy balance in constraining the timing of activity, which is primarily determined by the circadian clock [7,11–17]. Thus, differences in energy demand between field and laboratory conditions could be the fundamental feature ultimately leading to inversion in the timing of daily activity [7,11,17].

Subterranean rodents are excellent subjects to explore this proposition because their foraging activity in the field involves intense underground excavation, an intense energy demanding activity [18–23]. Moreover, the periodic day-time emergence of tuco-tucos to the surface [9,24] might indicate economy in thermoregulation costs, allowing allocation of the rest phase inside burrows during the coldest hours of the desert night [7,25].

Few studies have addressed continuous, 24h rhythms of metabolism in subterranean rodents [13, 26]. We set out to assess the daily patterns of metabolic rate and its interrelationships with body temperature (T_b_) and activity in wild-caught tuco-tucos. We hypothesized that tuco-tucos would show higher metabolic rates, levels of activity and T_b_ during darkness under laboratory light/dark cycles. Furthermore, we predicted that availability of a running wheel in the respirometry chamber would modulate amplitude but not timing of metabolic rate, as shown before for T_b_ [10]. To accomplish our investigation, we monitored individuals continuously for oxygen consumption (${\dot{\text{V}}\text{O}}_{2}$), T_b_, general motor activity and wheel-running over several consecutive days. Measurement of ${\dot{\text{V}}\text{O}}_{2}$ occurred inside a respirometry chamber, which, to our surprise, revealed a novel association between wheel-running and switches in timing of activity.

## Materials and Methods

### Ethics statement

All procedures followed the guidelines of the American Society of Mammalogists for the use of wild mammals in research [27] and the U.S. National Institutes of Health Guide for the Care and Use of Laboratory Animals [28]. All experiments were performed in Anillaco and were authorized by the Environmental Department of La Rioja (permits 028–10 and 062–08) and approved by the Ethics Committees of the Biosciences Institute of the University of São Paulo, Brazil (permit 164/2012), the University of Alaska Anchorage’s Institutional Animal Care and Use Committee (405977–1) and of the Faculty of Veterinary Sciences of La Plata National University, Argentina (permit 29-2-12).

### Animals

Tuco-tucos were trapped within a 3 km radius of the town of Anillaco (28° 48´ S; 66° 56´ W; 1350 m) in the ecoregion of the Monte Desert, Argentina. The animals were live-trapped within a 15km2 area surrounding the laboratory, with buried traps constructed from a 25-cm long PVC plumbing pipe with a 7.5-cm outer diameter. The traps were set by opening a burrow beneath a fresh mound of soil and positioning the pipe horizontally along the floor of the tunnel. Because the animals sometimes plugged the traps with loose soil, the traps were checked every 1–2 h, cleaned and reset as needed. Nine adult individuals (140–220 g) were used, of which five were females and four were males. Because these animals are solitary, they were housed individually in plastic cages (53×29×27 cm) equipped with running wheels (23 cm diameter, 10 cm wide, 1 cm between bars). Food (grass, native plants, carrot, sweet potato, rabbit pellets, oat, sunflower seeds) was provided *ad libitum* and replaced daily at various times. Water was not offered because subterranean rodents do not drink free water [19].

Cages were placed inside light-tight boxes equipped with one incandescent red light bulb providing continuous dim red light (1–5 lux) to facilitate animal care, and one fluorescent bulb of 200–250 lux at cage lid level connected to a timing device. Unless specified otherwise, tuco-tucos were kept under an LD cycle with 12 hours of “darkness” (1–5 lux) followed by 12 hours of light (LD 12:12), with lights on at 07:00 AM (local time, GMT -3).

Relative humidity ranged from 30–60% and room temperature was maintained at 25±2°C, which is within the thermoneutral zone of other *Ctenomys* species [29] (Tachinardi, unpublished). Data loggers (HOBO U10/003, Onset Computer Corporation, Bourne, MA) recorded room temperature and relative humidity every 15 minutes.

### Monitoring of wheel-running, general activity and body temperature

Tuco-tucos were surgically implanted with temperature sensitive transponders (G2 E-Mitters, Mini-Mitter, Bend, OR) to allow for continuous monitoring of core T_b_ and gross motor activity. Animals were anaesthetized using either ketamine/acepromazine (200 and 20 mg/Kg, respectively) or isoflurane anaesthesia (3%–5% with oxygen). Transponders were inserted into the peritoneal cavity through 1.5–2 cm vertical midline incision (1 cm bellow the rib cage) and sutured with poliglicolic acid thread (for more surgical details, see [10]). All surgeries were completed at least eight weeks prior to initiation of experiments.

Each cage was placed above a receiver (ER 4000, Mini-Mitter, Bend, OR) and data were collected and processed using the software VitalView (Mini-Mitter, Bend, OR); averages of T_b_ and activity were recorded each five minutes. Wheel-running was recorded as total revolutions in each 5-min interval by the ArChron Data Acquisition System (Simonetta System, Universidad Nacional de Quilmes, Buenos Aires, Argentina).

### Respirometry

Rates of O_2_ consumption were measured by open-flow respirometry during February and March of 2013 and 2014. In 2013, we used a FoxBox (Sable Systems, Las Vegas, NV) and Molecular Sieve 3Å (8–12 mesh, Sigma-Aldrich, Saint Louis, MO) as a desiccant with O_2_ measurement only. In 2014 we used the Field Metabolic System (Sable Systems, Las Vegas, NV) and a Nafion Dryer to remove moisture from the air [30]. Since ${\dot{\text{V}}\text{O}}_{2}$ data collected in the two years did not significantly differ (two-tailed t-test, p>0.05), we merged data from both years for further analysis.

During the experiments, animals were individually kept inside a respirometry chamber. This is the home cage (volume = 40L) with its wire lid replaced by a sealed clear acrylic lid, with fittings for in-flow and out-flow of air for the respirometry measures (S1 and S2 Figs). Outside air was pulled through the metabolic chamber at 450–650 mL/min, depending on the size of the animal. Before entering the chamber, outside air was passed through copper tubing (2m length) to facilitate equilibration of incurrent air temperature with air temperature of the animal room. Flow was generated by a vacuum pump and measured by a mass flow meter (part of the FoxBox System or the Mass Flow System-5, Sable Systems, Las Vegas, NV).

Excurrent air was drawn through Molecular Sieve 3Å or the Nafion dryer to remove moisture prior to measurements of gas concentrations. A subsample was passed through oxygen and carbon dioxide analyzers. The O_2_ analyzer was calibrated with ambient air every hour. Averages of flow rate and O_2_% were logged onto a computer each minute and corrected for baseline drift by linear interpolation using modified version of LabGraph [30].

Mass specific rate of oxygen consumption (mL g^−1^ h^−1^) was calculated using the following equations [30,31]:

$$V˙O_{2}=[V{˙}_{E}*\;\left(FIO_{2}–FEO_{2}\right)/\left(1–FIO_{2}*\left(1-RQ\right)\right)]/BM$$

${\dot{\text{V}}}_{\text{E}}$ = airflow exiting chamber (mL/min), FIO_2_ = fraction of O_2_ entering chamber, FEO_2_ = fraction of O_2_ exiting chamber, RQ = respiratory quotient (assumed to be 0.85, BM = body mass (Kg).

Integrity of the respirometry system was tested before the 2014 trials using alcohol burns [30].

Sufficient food for at least three days was placed inside the chamber at the beginning of the experiment. For trials lasting more than three days, additional food was supplied during the experiment by quickly opening and re-sealing the chamber. Chamber temperature was 25±1°C, recorded every 15 minutes by a data logger (HOBO U10/003, Onset Computer Corporation, Bourne, MA). Animals were weighed before and after each trial.

### Experiments

We performed continuous 5–9 day long respirometry trials for each animal, previously entrained by CE12:12, using two protocols. In the first (N = 4), respirometry trials were initiated without animal access to a running wheel and wheels were added on day three inside the chamber. In the second protocol (N = 5), trials started with a running wheel inside the chamber but removed on the third day. Activity and T_b_ were monitored continuously for at least 3 days before, during, and for 3 days after the respirometry trials.

### Data analysis

Animal activity and T_b_ were firstly depicted in double-plotted actograms using the software El Temps (Díez-Noguera, Universitat de Barcelona, Spain, 1999). Actograms allowed visual estimation of phase and rhythmic pattern.

To quantify phase changes in different conditions, we used a modified version of the diurnality index (D) proposed by Hoogenboom et al. [32][6,17]:

$$D=\sum \;{\left[\left(T_{L}-M\right)-\left(T_{D}-M\right)\right]}_{i}/\;\sum \;{\left[\left(T_{L-}M\right)+\left(T_{D}-M\right)\right]}_{i}$$

where T_Li_ and T_Di_ correspond respectively to each T_b_ measure during the light and dark phase (only values above the mean were considered) and M corresponds to the mean T_b_ during light and dark. This index is symmetric around 0 and runs from -1 (no high T_b_ during the day) to +1 (high T_b_ only during the day). We used T_b_ to calculate the D-Index because it was a variable recorded throughout all conditions.

${\dot{\text{V}}\text{O}}_{2}$ data are presented as means±SEM. We tested for the significance (α = 0.05) of differences in variables under different conditions using one-way ANOVAs (for multiple group comparisons) or two-tailed Student’s t-test (when only two conditions were compared). To test for significant associations among D-Indices and measured variables, we ran Pearson’s product-moment correlation tests. All analyses were performed with R version 2.11.1 [33].

## Results

**Fig 1:**
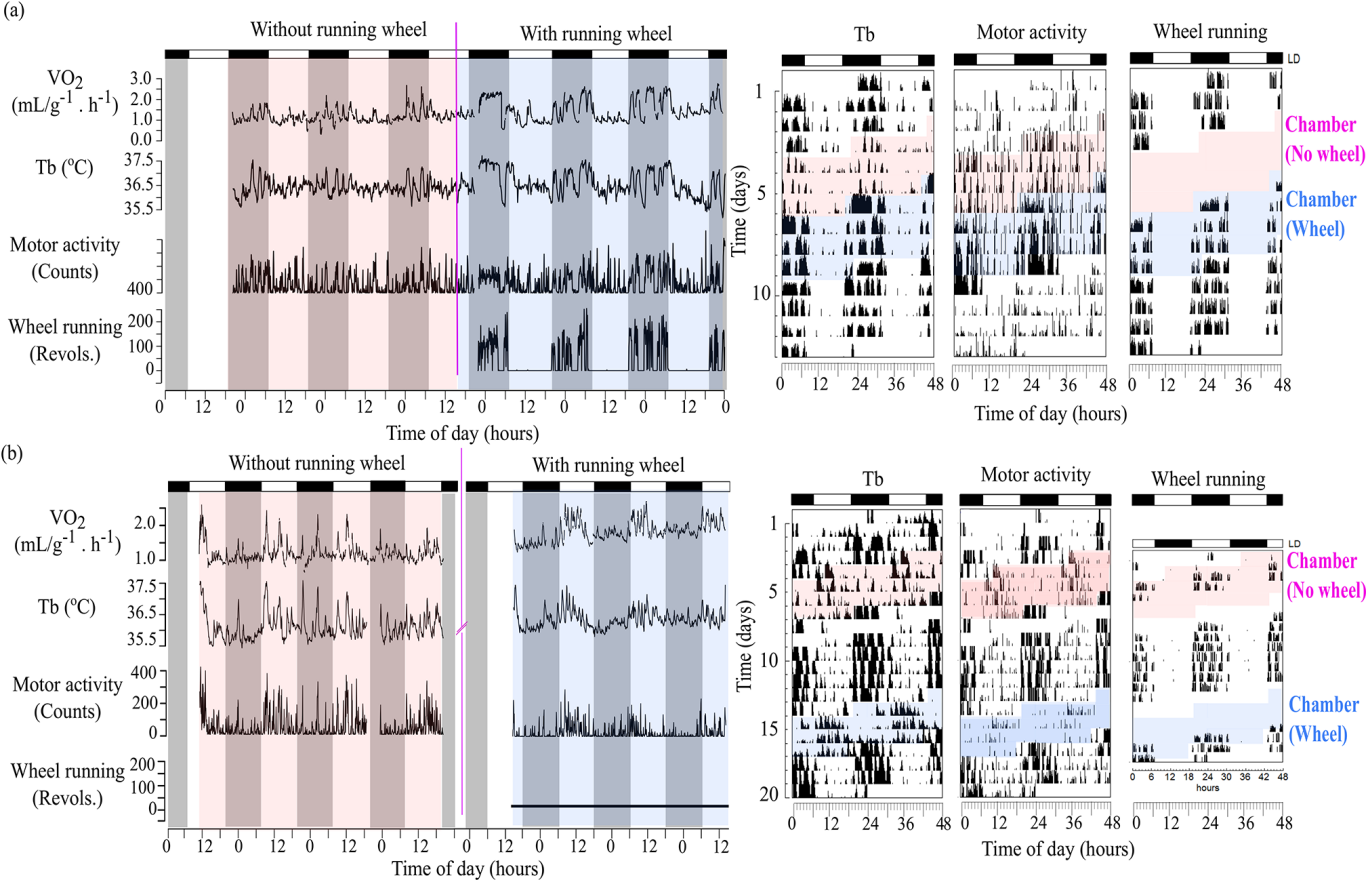
Simultaneous measurements of daily rhythms in oxygen consumption (${\dot{\text{V}}\text{O}}_{2}$), body temperature (T_b_), gross motor and wheel-running activity of tuco-tucos. Left: temporal series collected when the animal was inside the respirometry chamber, with and without a running-wheel. Shaded areas indicate dark phases and white areas light phases. Right: actograms across experimental conditions. Pink and blue backgrounds indicate data from animals inside the respirometry chamber, with and without access to wheels, respectively. (A) Representative individual that did not switch phase inside the respirometry chamber. Pink line in the left figure indicates introduction of the wheel to the chamber. (B) Representative individual that switched from nocturnal to diurnal inside the respirometry chamber. There was a 7-day interval outside the respirometry chamber before the wheel introduction due to technical problems. Pink broken line in the right figure separates days with and without wheels. General conditions: LD12:12 (L = 200–250 lux), 25±2°C and food *ad libitum*.

Before the start of the respirometry trials, all animals displayed a nocturnal pattern with high T_b_, general activity and wheel-running concentrated in the dark phase. When animals were placed into the respirometry chamber, some animals showed a radical and immediate change in their timing of peak ${\dot{\text{V}}\text{O}}_{2}$, T_b_ and general activity. While some (N = 3) remained clearly nocturnal (D-Indices<-0.4), the majority (N = 6) changed their rhythmic pattern and became either robustly diurnal (D-Indices>0; N = 3) or did not show clear nocturnality/diurnality (D-Indices between -0.1 and 0.1; N = 3). Fig 1A and 1B are representative data of one animal that remained nocturnal and of one that switched to diurnality inside the respirometry chamber, respectively. The left sections display time series of the variables inside the respirometer (colored backgrounds) to highlight their amplitude changes. The right sections display the corresponding whole data set (including days outside the chamber) in actogram format to highlight phase shifts. Because the respirometry measurement involved days with and without access to running wheels, blue and pink backgrounds indicate these two different conditions, respectively. As can be seen in Fig 1A, an animal that remained nocturnal showed highest values of all variables during the dark phase, including vigorous wheel-running episodes that appear clearly at the bottom of Fig 1A (left, blue background) and along the running-wheel actogram (right, blue background). On the other hand, Fig 1B shows data of an animal that switched to diurnality, as indicated by a shift of the highest values of variables to the light phase. This shift is more easily visualized in the right actograms, where timing of high amplitude Tb and motor activity switch to the light phase inside the colored sections, in dramatic contrast to the undisturbed patterns displayed in Fig 1A (right). Most notably, however, there was a total suppression of wheel-running activity in this animal, as indicated by no revolutions in the bottom of Fig 1B (left, blue background) and along the running-wheel actogram (right, blue background), again in contrast to the pattern exhibited in Fig 1A.

**Fig 2:**
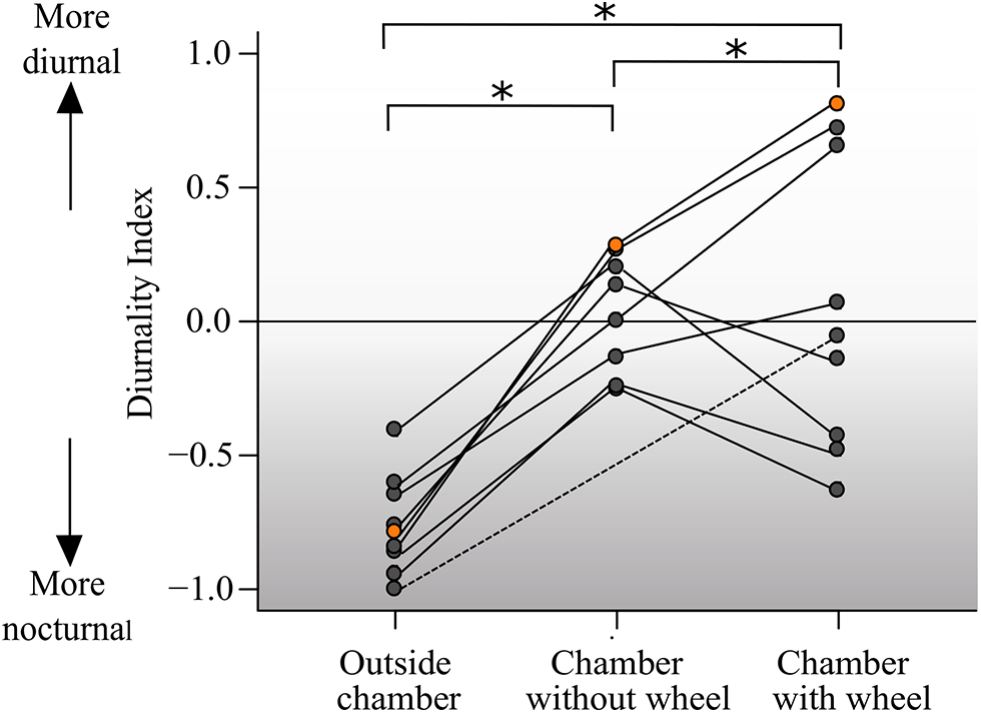
Variation of diurnality indices across the stages of the experiment. D-index for individual tuco-tucos (N = 9) along days outside and inside the respirometry chamber both with and without running-wheels. D-Index for each individual in the different conditions are connected by a line. Points in orange indicate the values for animal #143 which showed the highest discrepancy in D-Indices across conditions. Dashed line connects the values for animal #146, which was not submitted to the “chamber without wheel” condition.

D-indices ranged from -0.98 to -0.39 when animals were outside the respirometry chamber. Inside the respirometry chamber, D-indices ranged from -0.23 to +0.29 in the absence of the running wheel and from -0.61 to +0.83 when the wheel was available (Fig 2). One individual showed a particularly dramatic change in the D-Index, switching from -0.83 outside the chamber to +0.83 inside the chamber in the presence of the wheel (indicated by orange). Finally, data of one individual that was not measured without wheel was included, highlighted with a dashed line. Differences in D-Indices among the three conditions were statistically significant (p<0.001).

${\dot{\text{V}}\text{O}}_{2}$ followed the same rhythmic patterns as general activity and T_b_ (Fig 1). In addition to the daily variation, ${\dot{\text{V}}\text{O}}_{2}$ periodically peaked for episodes of more than one hour corresponding to bouts of high general activity and T_b_. Mean ${\dot{\text{V}}\text{O}}_{2}$ of tuco-tucos was 1.305± 0.073 mL g^-1^ h^-1^. Mean ${\dot{\text{V}}\text{O}}_{2}$ of females (1.235± 0.060 mL g^-1^ h^-1^, N = 5) and males (1.384± 0.151 mL g^-1^ h^-1^, N = 4) did not significantly differ (p>0.05). In S1 Table, we present the mean values of ${\dot{\text{V}}\text{O}}_{2}$ and T_b_ for each individual, during days with and without access to running wheels.

**Fig 3:**
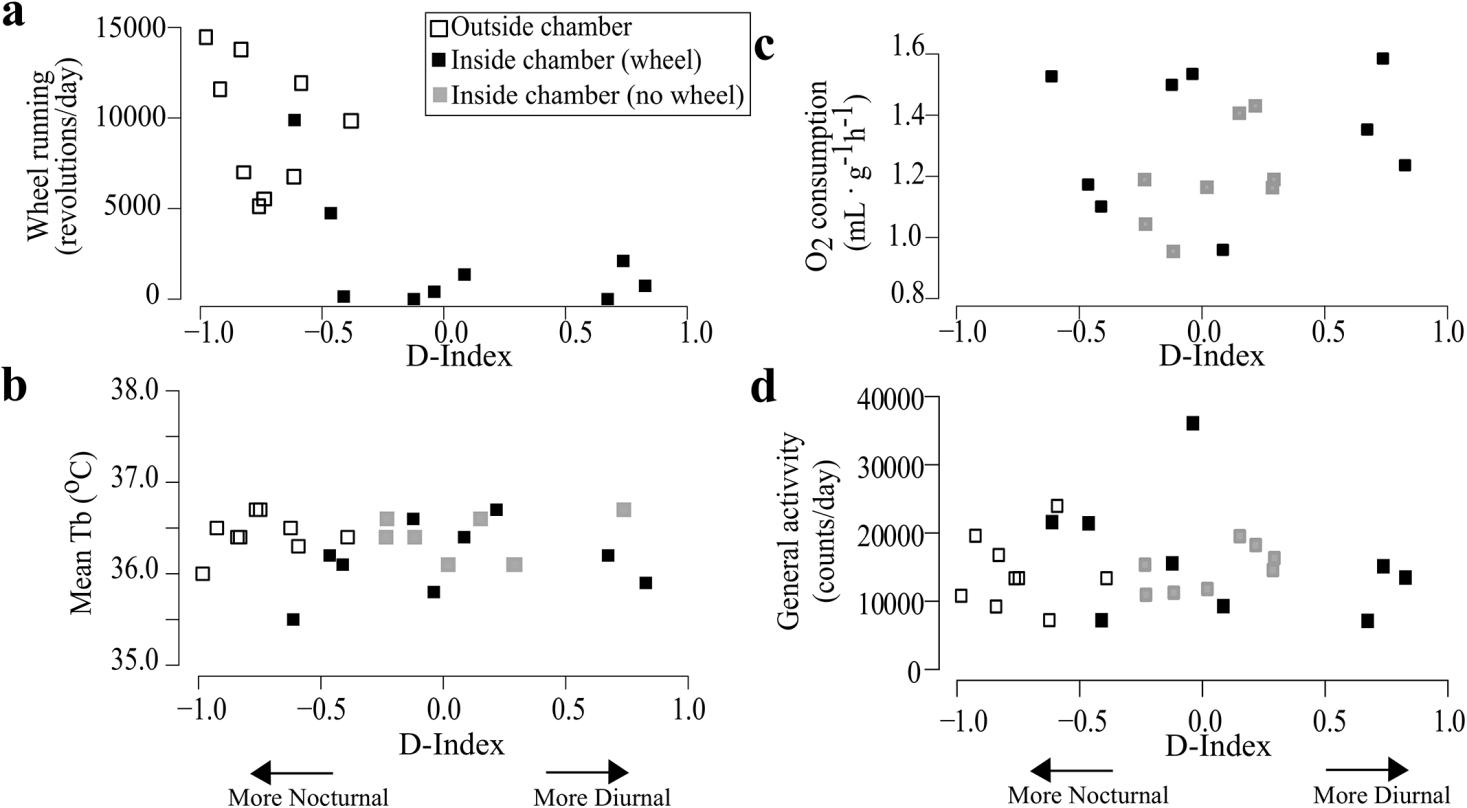
Wheel-running, mean T_b_ and mean Oxygen consumption of tuco-tucos in relation to diurnality indexes. Measurements for each individual (N = 9), across the stages of the experiment including days outside (white squares) and inside (black square) the respirometry chamber both with and without (grey squares) running-wheels. (A) Mean daily wheel-running levels are associated with nocturnality. (B) Mean body temperatures during each stage. There is no clear correlation with D-Indices. (C) Mean ${\dot{\text{V}}\text{O}}_{2}$ during each stage. There is no clear correlation with D-Indices. (D) Mean amount of general activity per day during each stage. There is no clear correlation with D-Indices.

Total amount of wheel-running revolutions per day was significantly reduced when animals were housed in the respirometry chamber (p<0.001). Whereas all individuals completed >5000 wheel revolutions/day outside the respirometry chamber, only one displayed such intense running while inside the chamber (Fig 3A). Daily amount of wheel-running correlated negatively with D-index (r = -0.73, p<0.001) with the most strongly nocturnal animals displaying the greatest amount of wheel-running (Fig 3A). Lower wheel-running and associated phase inversion occurred both in the animals exposed to the wheel immediately upon being placed in the respirometry chamber and in those animals that were provided a wheel after three days in respirometry chamber. Despite the drastic decrease in wheel-running activity, mean daily general activity, T_b_ and ${\dot{\text{V}}\text{O}}_{2}$ did not differ significantly among conditions (p>0.05) and neither correlated with D-Indexes (p>0.05) (Fig 3B, 3C and 3D, and S1 Table). Finally, no effect was observed in association to the order of wheel/non-wheel stages, in our protocol.

## Discussion

Despite showing day-time activity under field conditions, tuco-tucos consistently display nocturnal patterns when housed in the laboratory irrespective of access to running-wheels [8–10]. In the present study, we report the first displays of diurnality in the lab, which occurred exclusively during our respirometry experiment (Fig 1). Some individuals in the new environment of the sealed respirometry chamber completely suppressed running-wheel activity and switched to diurnality as revealed by T_b_, ${\dot{\text{V}}\text{O}}_{2}$ and general activity rhythms; while others remained nocturnal as usual in the laboratory and continued to run on the wheel (Fig 3).

The search for the critical factors which trigger the nocturnality/diurnality switch observed in other species often converge upon the issue of the meaning of the running-wheel activity in the laboratory [34–37]. In some rodent species, all individuals are diurnal in the field whereas in the laboratory some become nocturnal while others remain diurnal. Interestingly, when offered unrestricted access to running wheels, some diurnal individuals become nocturnal (grass rats, *Arvicanthis niloticus* [38]; degus, *Octodon degus* [39]; and mongolian gerbils, *Meriones ungiculatus* [4]). This phenomenon has been associated with a line of research devoted to investigating the “effect of” vigorous wheel-running on the period and phase of circadian activity rhythms [39–45]. Phase shifts in the free-running suprachiasmatic nuclei (SCN) due to “pulses” of intense running wheel activity are reported but are of very small magnitude [41,46]. Downstream from the SCN, however, wheel-running activity could act directly on the activity/inactivity signaling between the SCN and locomotor centers, as proposed by Kas and Edgar (1999). Their proposal was based on investigations of degus, a species that is known to switch phase from nocturnal to diurnal activity when provided access to a running wheel while in DD yet without any change to the basic free-running rhythm period.

The spontaneous suppression of wheel-running activity was displayed by all individuals that switched to diurnality (i.e., animals which showed D-Index>0.5) when exposed to the new environment of the respirometry chamber (Fig 3A). This phenomenon occurred in both of our trials in two consecutive years. It is noteworthy that general motor activity was maintained and switched to a diurnal pattern in all individuals that stopped running on the wheel (Fig 1). General activity levels did not change upon placement in the respirometry chamber, even in those animals that suppressed wheel-running. It is conceivable that this counterintuitive fact is due to the intensification of non-wheel as “digging-like” movements, intense grooming and other non-specific movements, which are generally observed when tuco-tucos are deprived of wheels.

**Fig 4:**
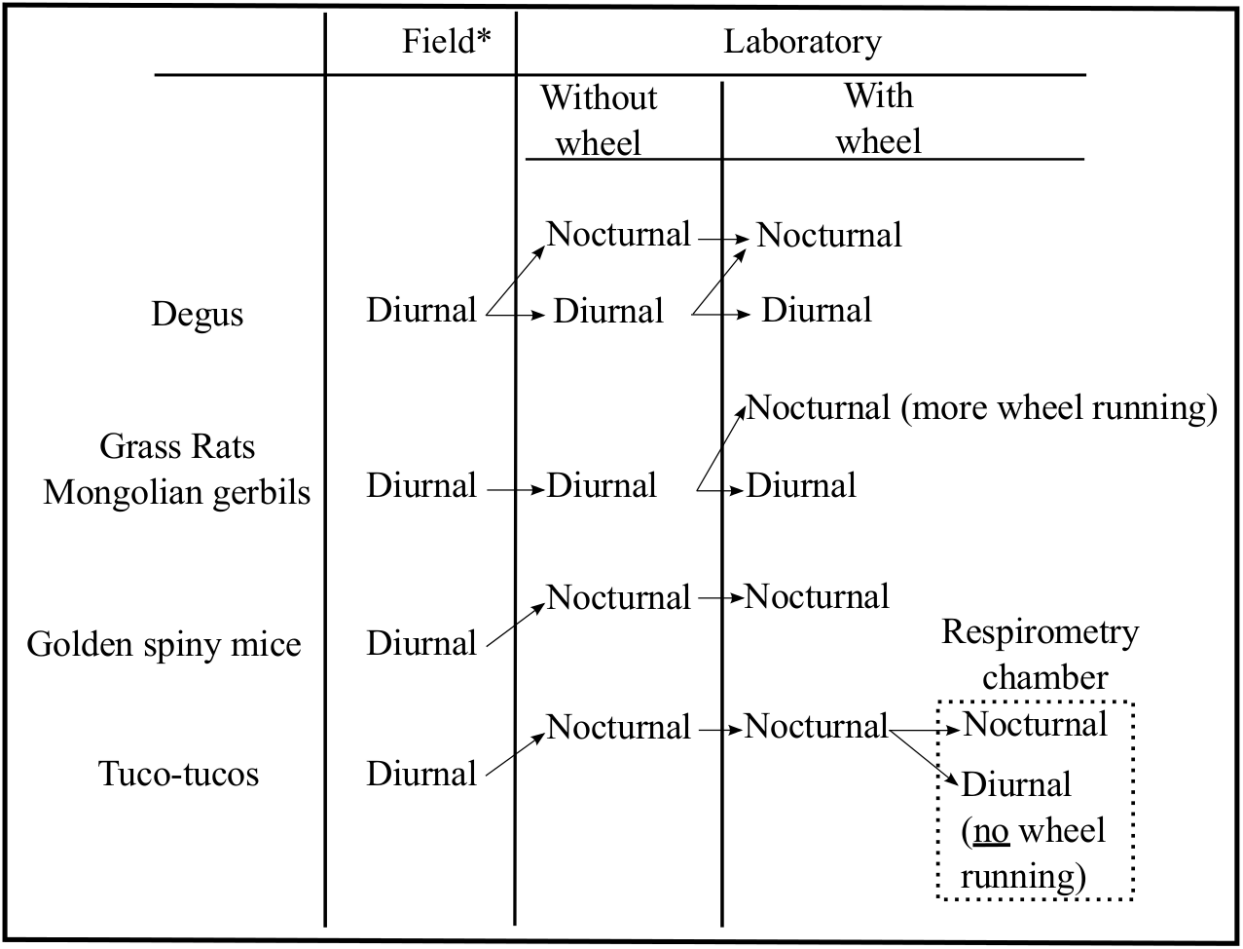
Schematic view of different phase switch patterns associated with the presence of running wheels. Based on: [1,39,56,57] for *Octodon degus*; [2,38] for *Arvicanthis niloticus*; [3,58] for *Acomys russatus*; [4] for *Meriones ungiculatus*; [9,10] for *Ctenomys* aff. *knighti*. *For each species, field data were collected using different methods and do not necessarily reflect activity patterns of whole populations.

Our finding of a phase inversion (nocturnal to diurnal) in tuco-tucos when housed within a respirometry chamber illustrates a novel association between running-wheels and timing of activity not observed in any of the previous work on degus, grass rats and Mongolian gerbils. In common with the above species, the greatest levels of activity are always associated with nocturnality (Fig 4). It has been proposed that a shift to nocturnality in response to elevated activity is associated to thermoregulation by consolidating the activity during a time of day when body temperature is naturally lower in diurnal species [39],[47–49]. However, this seems to be unlikely in tuco-tucos, based on our previous finding [10], that activity has a greater impact on body temperature during the dark phase, suggesting that the allocation of activity during the night would decrease, instead of enhance, heat loss.

Although phase inversion inside the respirometry chamber occurs concomitantly with suppression of wheel-running behavior, it is not “a response” to removal of the wheel (Fig 3) because when the wheel is removed from the respirometry chamber the nocturnal individuals do not switch to diurnality. Robust nocturnal patterns have been previously observed in 100% of 18 animals with wheels [8,9] and of 5 without wheels [9]. Additionally, wheel removal without switch in activity phase in tuco-tucos has been reported before in 100% of 6 animals, during experiments performed in other contexts [10], done in the same laboratory, under the same standard conditions (housing, feeding schedules, temperature, humidity).

Interestingly, both the most extreme diurnal and the most extreme nocturnal D-indices (Fig 2) are associated with the animals having access to a wheel while in the respiratory chamber. Because most animals didn’t actually use the wheel to run, this indicates that the wheel may also have a running-independent effect on the temporal pattern of activity. A small body of literature shows that the mere presence of the wheel in the environment can have behavioral and neurogenic effects [50]. Mice kept in an environment in which a locked wheel is present show less anxiety and enhanced fear memory than those kept in a cage without the wheel [51–52]. In our case, however, the suppression of running occurred spontaneously, in contrast to locking the wheel. This opens even more possibilities for future studies on running-independent effects of the wheel.

Diurnal/nocturnal switches in the laboratory occur so rapidly that it has been argued that this flexibility might represent an adaptive mechanism to sudden changes in the species’ natural environment [38, 39]. The switches in activity timing in tuco-tucos were triggered when inserted into a respirometry chamber (Fig 4) where tuco-tucos face mild alteration of gas composition of the ambient air (< 1% decrease in O_2_ and <0.5% increase in CO_2_). A survey of the literature of the effects of O_2_ and CO_2_ content of air on circadian patterns reveal mostly changes in amplitude, with rhythmic depression as a consequence of hypoxia or hypercapnia in rats [53,54]. However, minute phase changes have been observed in free-running golden hamsters exposed to pulses of hypoxic air [55]. These studies involved more extreme hypoxia/hypercapnia than faced by our tuco-tucos in the respirometry chamber and they were conducted with non-subterranean animals. It is conceivable that tuco-tucos, which live in sealed underground tunnels, are able to detect even small changes in gas composition and/or humidity. Perceived changes in the gas composition of the environment could serve as a triggering mechanism to incite an alertness response needed for predator avoidance or tunnel maintenance and, possibly, lead to changes in the temporal pattern of activity, as suggested by our results in the sealed chamber.

Several interesting insights have emerged from our simultaneous measurements of the interconnected ${\dot{\text{V}}\text{O}}_{2}$, T_b_, general motor and wheel-running rhythms. Our results clearly demonstrate that switches in timing of activity phase can occur concomitantly with spontaneous suppression of wheel-running. Apparently, in tuco-tucos activity timing and wheel-running associations appear in a reformulated perspective.

## Supporting Information

S1 Fig Respirometry chamber and schematic illustration of the experimental protocol. (A) Photography of the respirometry chamber without the running wheel. The chamber consists in a standard home cage with an acrylic lid with fittings to allow the airflow. The chamber was kept in a light-tight cabinet, which was the same used in the non-respirometry steps of the experiments. (B) Scheme of the experimental protocol. At first, the animal was kept in its home cage with access to a running-wheel. Then, it was placed in the respirometry chamber. One group was put in a chamber with running-wheel and the other in a chamber without a wheel. The group that started with the wheel would then have it removed, while the other would have the wheel added to the chamber. After the respirometry trials, measurements would continue in a standard home cage.
(DOCX)

S2 Fig Scheme of the respirometry system. (DOCX)

S1 Table Summary of the variables measured under different conditions, for each individual. ^1^In mL^.^ g^-1^h^-1^,represented as mean±SD. ^2^In °C. ^3^Mean total daily revolutions.
(DOCX)
