# Supplementary material for: Nocturnal to Diurnal Switches with Spontaneous Suppression of Wheel-Running Behavior in a Subterranean Rodent
Source: PLoS One. 2015 Oct 13;10(10):e0140500. doi: 10.1371/journal.pone.0140500 (PMC4603895; doi:10.1371/journal.pone.0140500)
Supplement: S1 Fig — (A) Photography of the respirometry chamber without the running wheel. The chamber consists in a standard home cage with an acrylic lid with fittings to allow the airflow. The chamber was kept in a light-tight cabinet, which was the same used in the non-respirometry steps of the experiments. (B) Scheme of the experimental protocol. At first, the animal was kept in its home cage with access to a running-wheel. Then, it was placed in the respirometry chamber. One group was put in a chamber with running-wheel and the other in a chamber without a wheel. The group that started with the wheel would then have it removed, while the other would have the wheel added to the chamber. After the respirometry trials, measurements would continue in a standard home cage. (DOCX) [file pone.0140500.s001.docx]

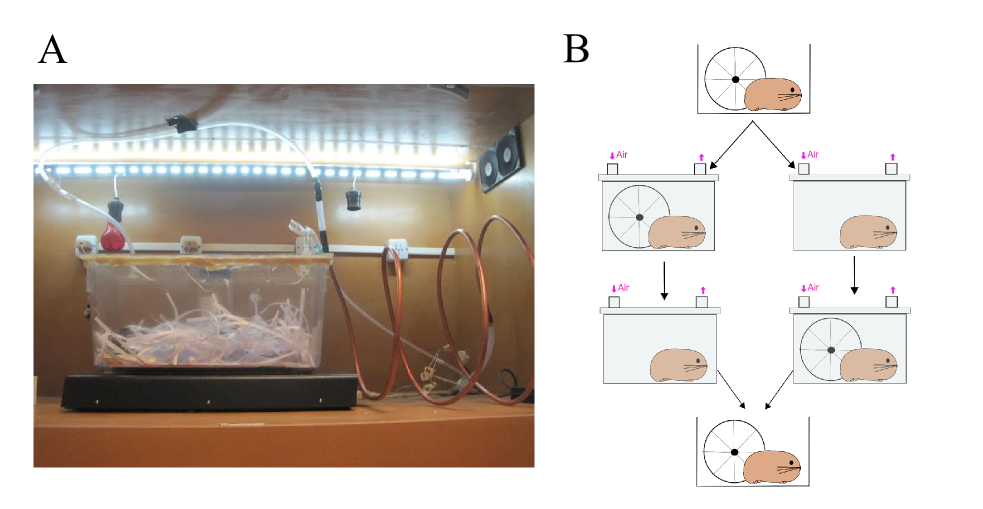


**Figure S1**. Respirometry chamber and schematic illustration of the experimental protocol. A. Photography of the respirometry chamber without the running wheel. The chamber consists in a standard home cage with an acrylic lid with fittings to allow the airflow. The chamber was kept in a light-tight cabinet, which was the same used in the non-respirometry steps of the experiments. B. Scheme of the experimental protocol. At first, the animal was kept in its home cage with access to a running-wheel. Then, it was placed in the respirometry chamber. One group was put in a chamber with running-wheel and the other in a chamber without a wheel. The group that started with the wheel would then have it removed, while the other would have the wheel added to the chamber. After the respirometry trials, measurements would continue in a standard home cage
