# Supplementary figures and images for: Nocturnal to Diurnal Switches with Spontaneous Suppression of Wheel-Running Behavior in a Subterranean Rodent
Source: PLoS One. 2015 Oct 13;10(10):e0140500. doi: 10.1371/journal.pone.0140500 (PMC4603895; doi:10.1371/journal.pone.0140500)

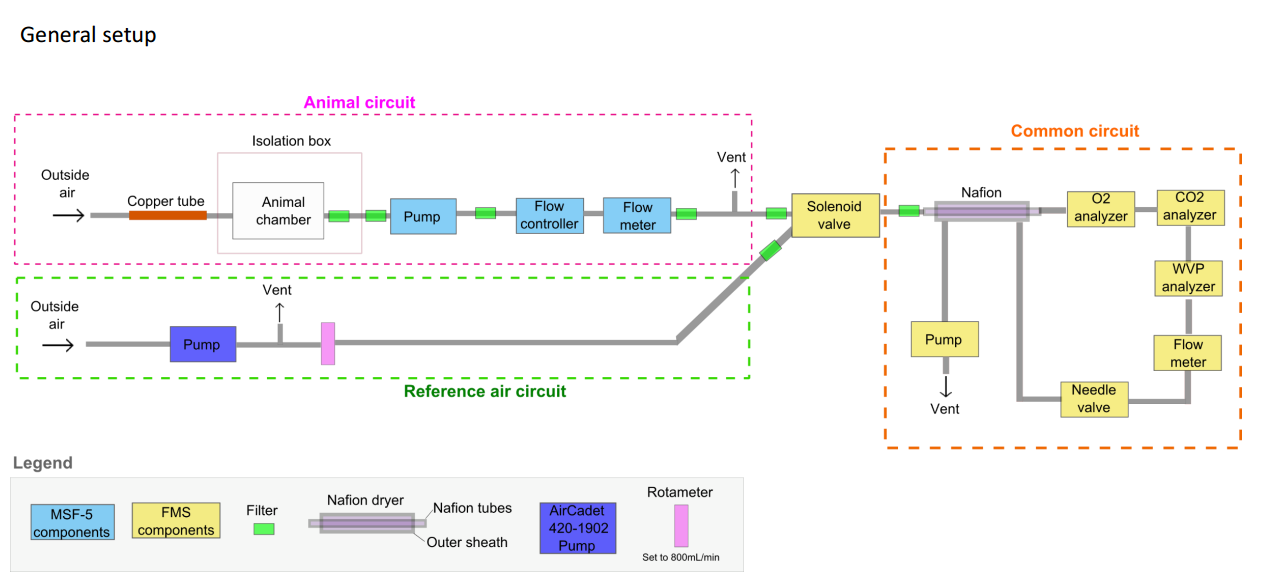


**Figure S2. Scheme of the respirometry system.**

Supplement: S2 Fig — (DOCX) [file pone.0140500.s002.docx]
