# Supplementary material for: Nocturnal to Diurnal Switches with Spontaneous Suppression of Wheel-Running Behavior in a Subterranean Rodent
Source: PLoS One. 2015 Oct 13;10(10):e0140500. doi: 10.1371/journal.pone.0140500 (PMC4603895; doi:10.1371/journal.pone.0140500)
Supplement: S1 Table — 1In mL. g-1h-1,represented as mean±SD. 2In °C. 3Mean total daily revolutions. (DOCX) [file pone.0140500.s003.docx]

**Table S1. Summary of the variables measured under different conditions, for each individual**. ^1^In mL ^.^ g^-1^h^-1^,represented as mean±SD . ^2^In ^o^C. ^3^Mean total daily revolutions.
